# Peak expiratory flow mediates the relationship between handgrip strength and timed up and go performance in elderly women, but not men

**DOI:** 10.6061/clinics/2016(09)06

**Published:** 2016-09

**Authors:** Raphael Mendes Ritti-Dias, Gabriel Grizzo Cucato, Fábio Gazelato de Mello Franco, Maysa Seabra Cendoroglo, Fábio Nasri, Maria Luiza Monteiro-Costa, José Antonio Maluf de Carvalho, Luciana Diniz Nagem Janot de Matos

**Affiliations:** Hospital Israelita Albert Einstein, São Paulo/SP, Brazil

**Keywords:** Elderly, Mobility, Strength, Pulmonary Function, Aging

## Abstract

**OBJECTIVE:**

The aim of the present study was to verify if there is sex difference in the associations among handgrip strength, peak expiratory flow (PEF) and timed up and go (TUG) test results.

**METHODS:**

The sample included 288 consecutive elderly men (n=93) and women (n=195). Functional capacity was measured using the TUG test, and muscle strength was measured based on handgrip. Moreover, as a measure of current health status, PEF was evaluated. Linear regression procedures were performed to analyze the relationships between handgrip and both PEF and TUG test results, with adjustment for confounders, and to identify the possible mediating role of PEF in the association between handgrip strength and TUG test results.

**RESULTS:**

In men, handgrip strength was associated with both PEF and TUG performance (*p*<0.01). After adjustment for PEF, the relationship between handgrip strength and TUG performance remained significant. In women, handgrip strength was also associated with both PEF and TUG performance (*p*<0.01). However, after adjustment for PEF, the relationship between handgrip strength and TUG performance was no longer significant.

**CONCLUSION:**

Mobility in the elderly is sex dependent. In particular, PEF mediates the relationship between handgrip strength and TUG performance in women, but not in men.

## INTRODUCTION

Many factors have been associated with loss of functionality during aging and it is well known that central and peripheral mechanisms can be involved in the process 1. Aging reduces muscle strength, power, and balance and also provokes modifications in the pulmonary system, such as thoracic stiffness, decreased pulmonary compliance and reduced respiratory muscle function 2. The sum of all these alterations is partially responsible for maintaining a vicious cycle of loss of muscle mass, which culminates in sarcopenia and fragility, in turn causing more mobility limitations and functional impairment 3.

The interrelationship between the pulmonary and the muscular systems has been described in the literature. Fragoso et al. 4 specifically verified that peak expiratory flow (PEF) is associated with a reduced ability to engage in the activities of daily life and increased mobility disability and death, independent of multiple potential confounders, such as age, smoking and chronic lung disease. In addition, Sillanpaa et al. 5 recently demonstrated that reduced mobility is directly regulated by a decline in muscle strength and power and is also partly mediated by decreases in pulmonary function. However, it is important to remember that aging affects neuromuscular 6,7 and pulmonary 8 function differently in men and women.

Although women already have lower muscle mass and smaller lungs than men, which predispose women to being at greater risk of physical incapacity 9, conversely, during aging, men exhibit greater loss of muscle strength 10 and respiratory function 11 than women. The most important clinical point in this context is the fact that the interrelationships among muscle strength, pulmonary function and mobility may be different between the sexes during aging, which may indicate the need for different clinical approaches. Thus, the identification of factors that link muscle strength with mobility could support the development of specific therapeutic interventions and could also be used to prevent or modify the emergence of mobility disability in elderly men and women in different ways. The aim of the present study was therefore to verify if there is a sex difference in the associations among handgrip strength, PEF and timed up and go (TUG) test results.

## MATERIALS AND METHODS

### Sample

The sample included 288 consecutive elderly men (n=93) and women (n=195) recruited at a geriatric hospital (Vila Mariana’s unit of the Hospital Israelita Albert Einstein, São Paulo – Brazil). This hospital is specialized in the care of elderly individuals over 65 years of age. The patients attended included institutionalized and community-dwelling elderly. In the unit, a multidisciplinary team (physicians, physiotherapists, psychologists, nurses, nutritionists and kinesiologists) provided optimal health and social support for the elderly subjects. Elderly were included if they were aged ≥65 years; had no evidence of chronic obstructive pulmonary disease; had no evidence of disability; and had no sign of dementia, as analyzed based on the clinical history and the Mini-Mental State Examination (score <15 points).

The procedures were in accordance with the ethical standards of the hospital’s Committee of Ethics for Analysis of Research Projects on Human Experimentation and with the Helsinki Declaration of 1975 (revised in 1983).

### Measures

Clinical characteristics were obtained from medical records and physical examination, and anthropometric measures were obtained by calculating body mass indices. Instrumental activities of daily living were assessed using the Lawton-Brody scale 12 and basic activities of daily living were analyzed using Katz’s questionnaire 13. Additionally, functional capacity was measured using the TUG test, and muscle strength was assessed based on handgrip. Finally, as a measure of current health status, PEF was evaluated, as detailed below.

### Mobility

The TUG test was applied to estimate mobility 14. The subjects were instructed to stand up from a chair without using their arms for support; to walk around a cone placed 3 m in front of the chair, moving as quickly as possible while taking care both not to run and to remain safe; and to return to the original sitting position. The time (in seconds) spent on performing this activity was recorded.

### Muscle strength

Muscle strength was measured via a handgrip test using a mechanical dynamometer (Saehan Corporation, 973, Yangdeok-Dong, Masan 630-728, Korea) following previous recommendations 15. During measurement, the subjects sat with their feet touching the ground, their elbows flexed to 90° and their forearms in the neutral position. All subjects performed three grips with their dominant hand, and the highest value was used for analysis.

### Peak expiratory flow

PEF was measured as an indicator of lung function. All subjects first received instructions about the use of a peak expiratory meter. During measurement, they were instructed to take a deep breath into the mouthpiece device (Respironics, Assess® Peak Flow Meter, Cedar Grove, New Jersey, U.S.A.) as vigorously possible. The neck stayed in the neutral position, rather than flexed or extended. After the point of full lung inflation, the patients breathed out with maximum effort into the peak flow meter. This procedure was performed twice and the highest value (L/min) was used for analysis.

### Statistical analysis

Descriptive statistics were performed using the frequency distribution and the mean ± standard deviation. To compare the characteristics between men and women, an independent t-test was conducted. Mediation was also tested using previously described procedures 16. Specifically, to test mediation, three linear regression equations were estimated: first, regression of the mediator (PEF) on the independent variable (TUG performance); second, regression of the dependent variable (handgrip) on the independent variable (TUG performance); and third, regression of the dependent variable (handgrip) on both the independent variable (TUG performance) and the mediator (PEF). To establish mediation, the following conditions must hold: the independent variable must be related to the mediator in the first equation, the independent variable must be related to the dependent variable in the second equation, and the mediator must affect the relationship between the independent and the dependent variables in the third equation. All analyses were adjusted for potential confounders (age, body mass index, smoking, coronary arterial disease and heart failure). Statistical significance was considered as *p*<0.05.

## RESULTS

The clinical characteristics and functional measures of the sample are presented in Table 1. Men were taller and heavier and presented a higher prevalence of coronary arterial disease and heart failure than women (*p*<0.05). The remaining variables, including handgrip strength, PEF and TUG performance, were similar between the sexes (N.S.).

**Figure 1:**
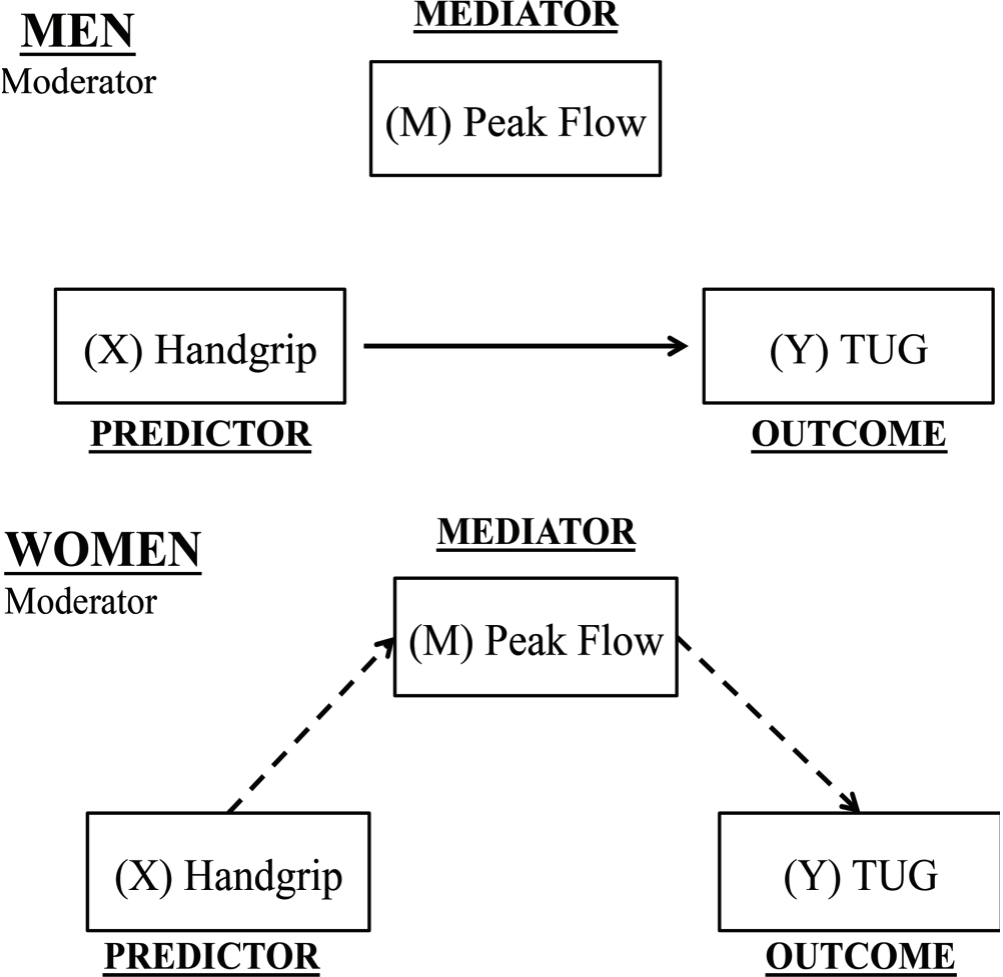
Summary of the results. (X) Predictor variable; (M) mediator; (Y) outcome. In men, the association between the predictor (handgrip) and the outcome (TUG performance) had a direct effect (unmediated). In contrast, in women, the association between the predictor (handgrip) and the outcome (TUG performance) was mediated by peak flow.

Table 2 presents the relationships among handgrip strength, PEF and TUG performance in men and women. In men, handgrip strength was associated with both PEF and TUG performance (*p*<0.01). After adjustment for PEF, the relationship between handgrip strength and TUG performance remained significant (Figure 1). In women, handgrip strength was also associated with both PEF and TUG performance (*p*<0.01). However, after adjustment for PEF, the relationship between handgrip strength and TUG performance was no longer significant (Figure 1).

## DISCUSSION

The main result of this study was that PEF mediates the relationship between handgrip strength and TUG performance in women, but not in men. Thus, mobility in elderly subjects is sex dependent.

Our results indicated that in both sexes, muscle strength was associated with mobility. Similar results were observed by Alley et al., who showed that handgrip strength was a strong predictor of mobility limitation in a representative sample of 9,897 men and 10,950 women 17. As handgrip strength measurement can be used as an indicator of general muscle strength among older subjects 14, the lower leg strength of elderly with lower handgrip strength 18 is the probable explanation for the link between handgrip strength and mobility.

Handgrip strength was also associated with PEF in both sexes. In a study conducted by Buchman et al. 19, which included separate proportional hazards models controlling for age, sex, and education, pulmonary function was associated with mobility, with a 1-unit lower level of pulmonary function at baseline associated with a 1.6-fold increase in the risk of developing mobility disability. The reasons for this association are obscure. However, handgrip strength has been consistently used as a sarcopenia indicator, providing information regarding the amount of muscle mass in the elderly 20. As PEF is influenced by respiratory muscle strength 21, reductions in respiratory muscle mass are likely to decrease PEF performance.

Mediation analyses have been used in behavioral and epidemiological studies in an effort to move beyond simple associations, which only analyze the relationship between dependent and independent variables and investigate potential mediators in a causal chain of clinical outcomes 16,22. Sillanpää et al. 5 observed that the decline in mobility with aging may be not only caused by decreases in both muscle strength and power but also mediated by decreases in pulmonary function. Although the reasons for this association are not completely understood, it has been suggested that respiratory muscle strength can lead to decreased pulmonary function. In turn, the inadequate energy supply caused by decreased pulmonary function could lead to impaired leg strength, contributing to the development of mobility disability, resulting in a vicious cycle.

The novelty of the results of the present study was that the mediation of the association between handgrip strength and TUG performance by PEF differed between the sexes. Whereas in women, PEF mediated the relationship between strength and mobility, in men, PEF did not present this mediating effect. As PEF can be interpreted as a surrogate marker of central function, a possible interpretation of this result is that central function plays an important role in the causal pathway between strength and mobility only in women. The reasons for this role of sex in the mediation by PEF is not clear, but certain hypotheses could be proposed. For example, studies have shown that decreases in muscle strength are faster in men than in women 6,7, so men may be more likely to present strength deficits in their extremities. Emphasizing this idea, in our sample, men presented a higher prevalence of cardiovascular diseases. Although it would be reasonable to hypothesize that heart failure and coronary disease cause more reduction in PEF and thus a more intense decrease in mobility and muscle strength, our results did not show this association. However, previous studies 23-25 have demonstrated that older women have higher susceptibility to pulmonary limitations during exercise than men. Together, all these results reinforce the idea that aspects of loss of functionality differ between men and women and that this loss is more peripheral in men.

The key point of this study was the importance of considering sex differences in clinical practice to allow more specific and individualized treatment of older people. In particular, because PEF is probably part of the causal chain between strength and mobility in women, its assessment could be more useful in elderly women. Moreover, respiratory muscle training presents positive effects in both sexes 26 but could be incorporated into the treatment of women earlier as a preventive measure.

This study has certain limitations. First, this was a cross-sectional study, which did not allow us to assess causality. Second, our sample presented different risk factors and we did not have information about the use of medication, which prevents future analysis of whether a relationship exists between these variables. Third, other variables not included in the current analysis may also play a mediating role in the relationship between handgrip strength and mobility. Future analyses including other potential mediators could enhance our knowledge of the relationship between handgrip strength and mobility in older adults.

In conclusion, the results of this study indicated that mobility in the elderly is sex dependent and that PEF mediates the relationship between handgrip strength and TUG performance in women, but not in men.

## AUTHOR CONTRIBUTIONS

Ritti-Dias RM, Cucato GG and Franco FGM were responsible for the study design. Carvalho JA, Cendoroglo MS, Monteiro-Costa ML and Nasri F were responsible for the data collection. Ritti-Dias RM, Cucato GG, Matos LD and Cendoroglo MS were responsible for the critical review of the manuscript.

## Figures and Tables

**Table 1:** Clinical characteristics and functional measures of the sample.

	Men (n=93)	Women (n=195)	*p*
Age, yrs	75.6 (6.9)	75.5 (7.9)	0.93
Height, m	1.54 (0.10)	1.59 (0.09)	<0.01
Weight, kg	63.3 (15.3)	73.0 (14.5)	<0.01
Body mass index, kg/m^2^	27.7 (4.7)	29.0 (5.1)	0.08
Handgrip strength, kgf	20.1 (12.1)	20.9 (9.2)	0.53
Peak expiratory flow, L	234 (160)	239 (122)	0.76
Timed up and go, sec	0.45 (0.49)	0.50 (0.48)	0.42
Lawton-Brody scale, score	25.5 (3.0)	25.5 (2.9)	0.80
Katz, score	5.8 (0.5)	5.8 (0.5)	0.99
Current smoking, %	9.7	10.8	0.16
Peripheral arterial disease, %	5.4	2.6	0.22
Coronary arterial disease, %	16.1	7.7	0.04
Heart failure, %	16.1	6.2	<0.01

The data are presented as the mean ± standard deviation and frequency.

**Table 2:** Linear regression between handgrip strength, peak expiratory flow and timed up and go performance in men and women.

	b	*p*
**Men**		
(Equation 1) Handgrip *vs*. Peak expiratory flow*	0.310	<0.01
(Equation 2) Handgrip *vs*. Timed up and go*	-0.497	<0.01
(Equation 3) Handgrip *vs*. Timed up and go **	-0.436	<0.01
**Women**		
(Equation 1) Handgrip *vs*. Peak expiratory flow*	0.596	<0.01
(Equation 2) Handgrip *vs*. Timed up and go*	-0.184	0.02
(Equation 3) Handgrip *vs*. Timed up and go **	-0.120	0.28

* Adjusted for age, body mass index, smoking, coronary arterial disease and heart failure.

** Adjusted for age, body mass index, smoking, coronary arterial disease, heart failure and peak expiratory flow.
